# *Legionella* 5S rRNA PCR melting temperature analysis discriminates high-risk species associated with disease severity

**DOI:** 10.1128/jcm.00356-26

**Published:** 2026-05-29

**Authors:** Aaron M. Pulsipher, Georges Khattar, Emily Harris, Valerie White, Camron Stout, Holenaraspur R. Vikram, Robin Patel, Patricia J. Simner

**Affiliations:** 1 Department of Critical Care, Mayo Clinic, Phoenix, Arizona, USA; 2 Division of Clinical Microbiology, Mayo Clinic, Rochester, Minnesota, USA; 3 Division of Infectious Diseases and Transplant Center, Mayo Clinic, Phoenix, Arizona, USA; 4 Division of Public Health, Infectious Diseases, and Occupational Medicine, Mayo Clinic, Rochester, Minnesota, USA; National Institute of Allergy and Infectious Diseases Division of Intramural Research, Bethesda, Maryland, USA

**Keywords:** clinical outcomes, melting temperature, PCR, diagnostics, *Legionella*

## Abstract

*Legionella* species are important causes of community-acquired pneumonia. The diagnostic performance and clinical implications of a fluorescence resonance energy transfer (FRET)-based real-time PCR assay targeting the *Legionella* 5S rRNA gene were evaluated, with emphasis on melting temperature (Tm) analysis to differentiate detected species and associated clinical outcomes. A retrospective multicenter cohort study of adults with laboratory-confirmed *Legionella* infection between 2019 and 2025 was conducted. PCR crossing point (Cp) and Tm data were analyzed and compared with species identified in culture from respiratory specimens. Associations between Tm and clinical outcomes were assessed using multivariable logistic regression. Among 51,489 PCR tests performed, 823 (1.6%) were positive. Among 376 *Legionella* infections, PCR demonstrated the highest diagnostic yield. Among patients undergoing all three diagnostic tests, the addition of PCR increased detection from 49% with urine antigen and culture combined to 100% (*P* < 0.001). Of 189 PCR-positive patient specimens with available Tm data, Tm showed a bimodal distribution, being either <66°C or ≥66°C. A Tm cutoff of ≥66°C discriminated *L. pneumophila*/*L. longbeachae* from other *Legionella* species with excellent performance (AUC, 1.00). Overall, 29.6% of infections had a Tm <66°C, consistent with non-*pneumophila/*non-*longbeachae Legionella* species. Infections due to *L. pneumophila*/*L. longbeachae*, which correspond to high Tm values in the study assay, were independently associated with ICU admission (adjusted odds ratio, 2.85; 95% CI, 1.33–6.11; *P* = 0.007), but not 90-day mortality. Real-time PCR targeting the *Legionella* 5S rRNA gene provides superior detection compared to culture and/or urine antigen testing and enables higher-risk species discrimination through Tm analysis.

**IMPORTANCE:**

Nearly one-third of *Legionella* infections in this cohort were attributable to non-*pneumophila* species, highlighting limitations of diagnostic strategies reliant on *Legionella* urine antigen testing or *L. pneumophila* PCR. The findings suggest that species-level inference can be derived from melting-temperature data generated during routine PCR testing. Incorporating validated melting-temperature interpretation into reporting workflows may improve epidemiologic surveillance and clinical understanding of characteristics of *Legionella* pneumonia.

## INTRODUCTION

*Legionella* is the sole genus within the family *Legionellaceae*, comprising 67 recognized species and three subspecies (1). Clinically significant species include *Legionella pneumophila*, *Legionella micdadei*, *Legionella bozemanii*, and *Legionella longbeachae*, among others. Globally, *L. pneumophila* has historically been considered to account for more than 90% of *Legionella* pneumonia, with *L. longbeachae* reported to predominate in Australia and New Zealand (2–4).

*Legionella* species most commonly cause community-acquired pneumonia, with extrapulmonary manifestations possible, but rarely occurring (4, 5). Diagnosis depends on clinical suspicion supported by laboratory testing. The *Legionella* urinary antigen test is widely used because of its ease of use, rapid turnaround, and high specificity (>95%), but it detects *L. pneumophila* serogroup 1 and may miss infections caused by other *L. pneumophila* serogroups and non-*pneumophila Legionella* species (6). Culture on selective buffered charcoal yeast extract (BCYE) agar allows species identification and epidemiologic typing, but requires specific ordering (separate from routine respiratory cultures) and prolonged incubation (engendering a long turnaround time), limiting use in routine practice (7).

Nucleic acid amplification tests, including PCR, offer high sensitivity and can detect a range of species when broad-range targets such as ribosomal genes are used (8). Targeting the 16S rRNA gene permits species discrimination when sequencing is performed, while *L. pneumophila* is commonly detected using the macrophage infectivity potentiator (*mip*) gene and is included on some FDA-cleared multiplex pneumonia panels (9, 10). Metagenomic approaches, such as plasma cell-free DNA testing, may detect *Legionella* species (11). Molecular detection reflects DNA presence and may not distinguish active infection from contaminating DNA from reagents or sample collection, which may occur as *Legionella* species are environmental organisms present in water. Serology may aid in retrospective diagnosis but is not useful for acute clinical decision-making.

We recently described a multicenter cohort of patients with *Legionella* pneumonia, demonstrating substantial critical illness and short-term mortality. Diagnostic analysis from that study suggested that reliance on *Legionella* culture or urine antigen testing may fail to identify a substantial proportion of cases, with PCR demonstrating the highest positivity rate (12).

A laboratory-developed test (LDT) PCR assay targeting the *Legionella* 5S rRNA gene and using fluorescence resonance energy transfer (FRET) hybridization probes for product detection has been in use in our clinical practice since 2009. Although the assay is clinically validated for genus-level reporting, melting temperature (Tm) analysis, performed as part of the assay, provides information as to the detected species (8, 13). In this study, PCR Tm data associated with the assay were analyzed in relation to culture-confirmed species identification, patient characteristics, and clinical outcomes.

## MATERIALS AND METHODS

### Patient population

A retrospective multicenter cohort study of adults (≥18 years) with laboratory-confirmed *Legionella* pneumonia across Mayo Clinic sites (Rochester, MN; Jacksonville, FL; Scottsdale, AZ; Mayo Clinic Health Systems sites) between 1 January 2019 and 31 December 2025 was performed. The study was approved by the Mayo Clinic Institutional Review Board with a waiver of informed consent. Laboratory-confirmed infection was defined as any of the following: A positive *Legionella* urinary antigen test (BinaxNOW); a positive LDT *Legionella* PCR or detection of *L. pneumophila* by the BIOFIRE FILMARRAY Pneumonia (PN) Panel performed on sputum, tracheal secretions, or bronchoalveolar lavage (BAL) fluid; or a positive sputum, tracheal secretions, or BAL fluid culture on BCYE agar, with or without polymyxin B, anisomycin, and vancomycin, incubated for up to 14 days. Urine antigen testing and *Legionella* culture were performed locally at each site. Clinical data were manually abstracted into REDCap and included demographics, comorbidities, laboratory values, imaging findings, diagnostic modality, and clinical course (14). The primary outcome was 30-day mortality with a secondary outcome of needing intensive care unit (ICU) admission at any point during the index hospitalization (12).

### PCR assays

All sites used the same PCR assay, performed centrally at Mayo Clinic in Rochester, MN. The assay is a FRET probe real-time PCR targeting the 5S rRNA gene (https://www.mayocliniclabs.com/test-catalog/Overview/89564#performance). During clinical validation, inclusivity testing utilizing American Type Culture Collection/National Collection Type Culture strains was completed with 15 serogroups of *L. pneumophila* (*L. pneumophila* serogroups 1–14, 15/16 - Tm: 68.4) and 18 additional *Legionella* species (*Legionella anisa* - Tm: 61.6; *L. bozemanae* - Tm: 61.4; *Legionella dumoffii* - Tm: 61.2; *Legionella gormanii* - Tm: 68.3; *Legionella jordanis* - Tm: 63.8; *L. longbeachae -* Tm: 68.6, *L. micdadei* - Tm: 63.1; *Legionella oakridgensis -* Tm: 63.2; *Legionella hackeliae* - Tm: 53.6; *Legionella maceachernii -* Tm: 63.1; *Legionella parisiensis -* Tm: 61.4; *Legionella sainthelensi -* Tm: 61.7; *Legionella cincinnatiensis -* Tm: 61.6; *Legionella lansingensis -* Tm: 56.2; *Legionella rubrilucens -* Tm: 47.8/55.9; and *Legionella wadsworthii -* Tm: 61.0). Two uncommonly encountered species of *Legionella*, *L. birminghamensis* and *L. feeleii,* were not detected by the assay during validation. In addition to the LDT PCR assay, one site utilized the BIOFIRE FILMARRAY Pneumonia (PN) Panel that included *L. pneumophila* among its targets. For patients undergoing PCR testing with the LDT PCR, specimen source, collection date, report date, crossing point (Cp), and Tm were collected and, when available, culture-confirmed species were compared to PCR results. The LDT PCR assay defined positivity as amplification with Cp values ≤ 35 (2019–2024) or ≤40 (2024–2025), with melting temperatures (Tm) consistent with *Legionella* species and fluorescence output ≥1.000. Samples with Cp values > 40 but acceptable Tm were confirmed by repeat testing and 16S rRNA PCR with sequencing prior to reporting. For statistical analysis purposes, Cp values > 40 were assigned a value of 40.

### Statistical analysis

The area under the receiver operating characteristic curve (AUC) was calculated using Tm to assess discrimination between species with Tm values ≥ 66°C (*L. pneumophila* and *L. longbeachae*) and those with Tm values <66°C (non-*pneumophila*, non-*longbeachae* species) among culture-confirmed cases.

Continuous variables were reported as medians with interquartile ranges (IQR), with categorical variables reported as counts with percentages. Comparisons between groups were conducted via Fisher’s exact test for categorical variables and Wilcoxon rank-sum test for continuous variables. Univariate and multivariable logistic regression were performed for PCR detections with Tm <66°C and ≥66°C to estimate adjusted associations with 30-day mortality and ICU admission. Variables chosen were based on significant baseline differences between groups (BMI and hypertension), as well as those that demonstrated significant association in our entire cohort analysis (age and immunocompromised status) (12).

Among patients who underwent urine antigen testing, culture, and PCR, the diagnostic yield of individual and combined testing strategies was calculated by assessing the proportion of cases detected with each test. Incremental yield associated with the addition of PCR was assessed using paired comparisons and McNemar’s test. Absolute differences in detection rates were derived from within-patient discordant results.

Kaplan–Meier survival curves for 90-day survival were generated for the Tm <66°C and Tm ≥66°C groups as a binary variable, with differences assessed using the log-rank test. All analyses were conducted using Stata version 19.5 (StataCorp). Two-sided *P*-values <0.05 were considered statistically significant.

## RESULTS

### Diagnostic ordering practices

Urine antigen alone was the most frequently ordered diagnostic test (37.8%), followed by urine antigen plus culture plus PCR (26.1%) and culture plus PCR (18.4%) (Table 1). Of 213 PCR-positive respiratory specimens, 189 were positive by the LDT PCR, 32 by the multiplex pneumonia panel, and eight by both the LDT PCR and multiplex pneumonia panel. Among 89 patients with positive culture results, 76 also underwent PCR testing, of whom 73 (96.1%) had positive PCR results. The three patients with culture-positive but PCR-negative results were infected with *L. pneumophila*, *L. longbeachae*, and *L. anisa*. Additionally, among 161 patients with positive PCR results who underwent *Legionella* culture, 73 (45.3%) had positive culture results.

**TABLE 1:** *Legionella* diagnostics ordered and diagnostic yield in confirmed *Legionella* cases

Test combinations	Observed testing pattern (*n*)	Estimated diagnostic yield (%)^a^
Urine antigen only	142 (37.8%)	22 (22.5%)
Culture only	3 (0.8%)	40 (40.8%)
PCR only	22 (5.9%)	94 (95.9%)
Urine antigen and PCR	30 (8.0%)	98 (100%)
Urine antigen and culture	12 (3.2%)	48 (49.0%)
Culture and PCR	69 (18.4%)	95 (96.9%)
Urine antigen, culture, and PCR	98 (26.1%)	98 (100%)

^a^ Diagnostic yield was calculated among patients who underwent all three tests (i.e., urine antigen, culture, and PCR), estimating the proportion of cases that would have tested positive if only the specified test combination had been ordered.

Among patients undergoing all three diagnostic tests (*n* = 98), addition of PCR (multiplex pneumonia panel or LDT PCR) increased detection compared to urine antigen alone (22.4 *versus* 100%; absolute increase 77.6%, *P* < 0.001; Table 2) and culture alone (40.8 *versus* 96.9%; absolute increase 56.1%, *P* < 0.001). When both urine antigen and culture were performed together, inclusion of PCR increased detection from 49.0% to 100% (absolute increase 51.0%, *P* < 0.001).

**TABLE 2:** Incremental diagnostic yield of *Legionella* testing strategies

Strategy comparison	Discordant pairs^a^ (*n*)	Absolute increase (%)	McNemar *P*-value
Urine antigen *versus* urine antigen plus PCR	76	77.6%	<0.001
Culture *versus* culture plus PCR	55	56.1%	<0.001
Urine antigen plus culture *versus* urine antigen plus culture plus PCR	50	51.0%	<0.001

^a^ Discordant pairs represent patients negative by the first strategy (urine antigen and/or culture) and positive after addition of PCR.

### PCR testing volume and positivity

**Fig 1:**
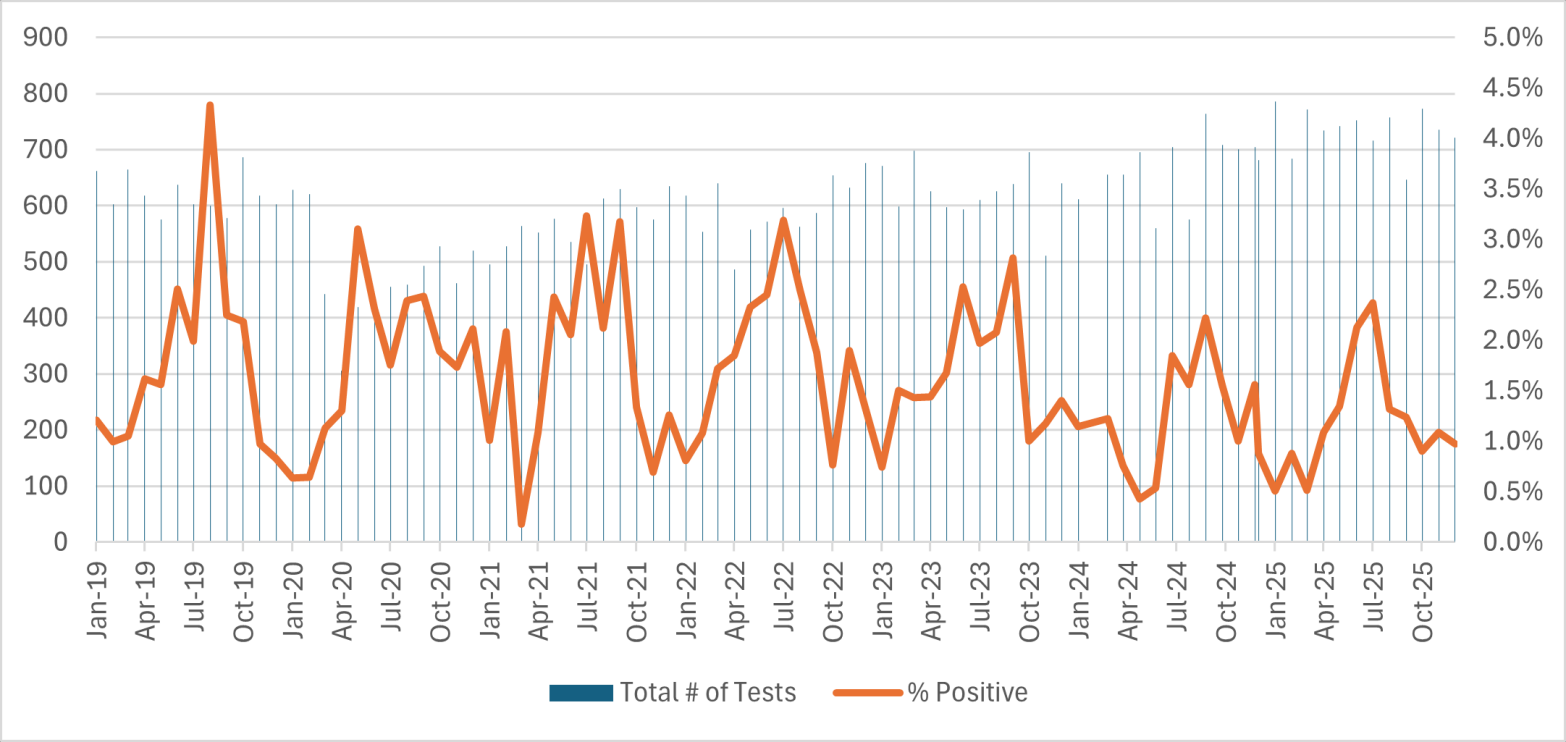
*Legionella* PCR test numbers (blue bars) and percent positivity (orange line) from January 2019 through December 2025.

Between 1 January 2019 and 31 December 2025, 51,489 LDT PCR tests were performed for both Mayo Medical Laboratory clients and Mayo Clinic patients, with 823 positive results (1.6% positivity; Fig. 1). Positivity demonstrated a seasonal pattern, peaking in summer months. PCR testing volume increased over time (coefficient 27.6 per year; 95% CI, 19.8–35.5; *P* < 0.001), while percent positivity showed a downward trend (slope −0.093% per year; 95% CI, −0.173% to −0.012%; *P* = 0.02).

Among 376 Mayo Clinic patients diagnosed with *Legionella* infection, 189 were positive by the LDT PCR assay and therefore had available Cp and Tm data. Median Cp was 33.3 (range 21.07 to >40). Culture-positive specimens had lower median Cp values than culture-negative/PCR-positive specimens (29.2 *versus* 35.7 cycles; *P* < 0.001). Patients with a negative urine antigen test had higher median Cp values than those with a positive urinary antigen test (33.5 *versus* 28.8 cycles, respectively; *P* = 0.006).

### Melting temperature and species discrimination

**Fig 2:**
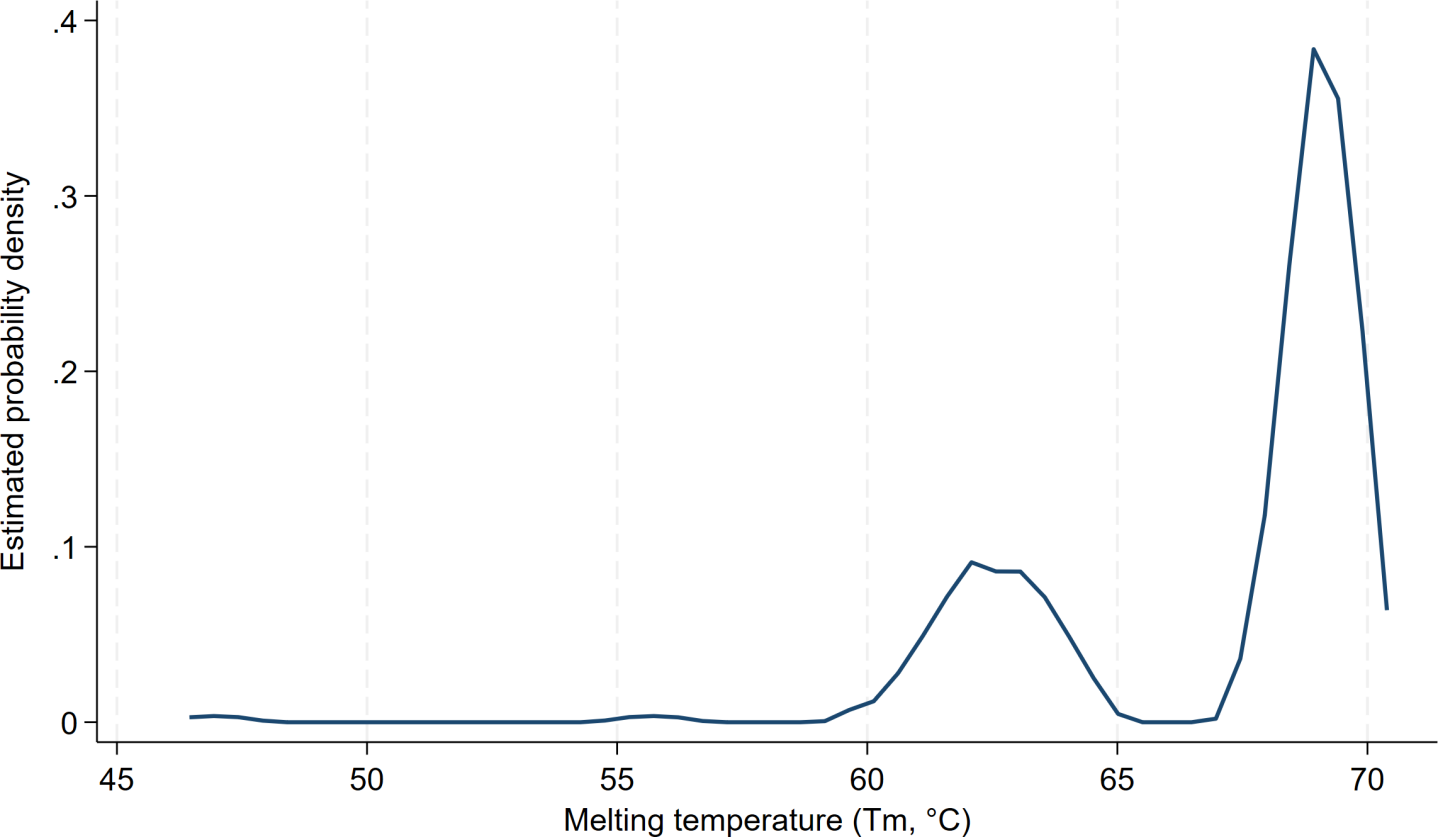
Distribution of melting temperatures observed by *Legionella* fluorescence resonance energy transfer probe-based PCR Kernel density estimate of melting temperatures (Tm) for all PCR-positive cases (*n* = 169). The y-axis represents the estimated probability density, such that the total area under the curve equals 1. A bandwidth of 0.5°C was used to improve visualization of the bimodal distribution. The distribution demonstrates one Tm cluster centered near 69°C with another centered near 62–64°C.

**Fig 3:**
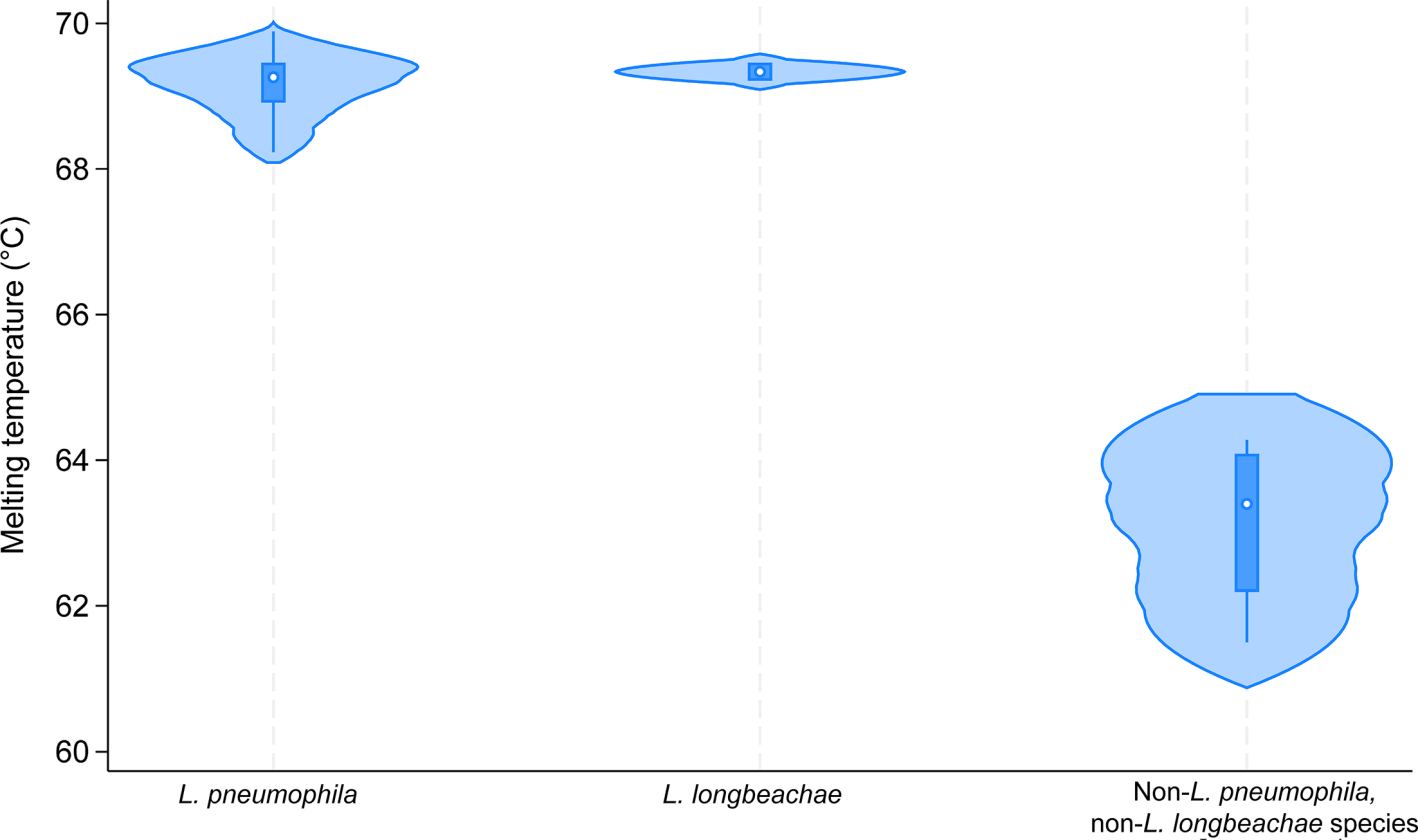
Melting temperature correlation with species identified by culture.

Tm values demonstrated a bimodal distribution (Fig. 2). The overall median Tm was 68.8°C (IQR 63.67–69.30°C), consistent with two distinct melting clusters. Among 72 culture-confirmed cases, Tm ≥66°C corresponded exclusively to *L. pneumophila* (96.8%) or *L. longbeachae* (3.2%) (Fig. 3). ROC analysis demonstrated excellent discrimination (AUC 1.00).

Overall, 56 patients (29.6%) had Tm values < 66°C and 133 (70.4%) Tm values ≥ 66°C, suggesting that nearly one-third of PCR-positive infections were due to non-*pneumophila/*non-*longbeachae* species.

### Clinical characteristics

As shown in Table 3, age and biologic sex were similar between patients with Tm values < 66°C and ≥66°C. Patients with Tm values ≥ 66°C had a higher median body mass index (27.2 *versus* 24.1, *P* < 0.001) and higher rates of hypertension (63.2 *versus* 41.1%, *P* = 0.006). They were also more likely to require hospital admission (89.5 *versus* 71.4%; *P* = 0.004) and ICU care (43.6 *versus* 19.6%; *P* = 0.002; Table 4). Overall oxygenation requirements differed between groups (*P* = 0.003), with mechanical ventilation being more frequent in those with Tm ≥66°C (36.1 *versus* 12.5%). Admitted patients with Tm ≥66°C had higher median SOFA-2 scores (3 *versus* 1, *P* = 0.01) (15). Hospital length of stay, ICU length of stay, new need for dialysis, and in-hospital and 30- and 90-day mortality did not differ between those with Tm values < 66°C and ≥66°C.

**TABLE 3:** Patient demographics stratified by *Legionella* PCR melting temperature (Tm, <66°C *versus* ≥66°C)

Variable	Tm <66°C	Tm ≥66°C	*P*-value
N	56	133	
Age (interquartile range)	68.3 (57.9–75.1)	65.6 (57.3–74.0)	0.53
Male	30 (53.6%)	68 (51.1%)	0.87
Body mass index (interquartile range)	24.1 (21.5–26.3)	27.2 (23.0–30.7)	<0.001
Hypertension	23 (41.1%)	84 (63.2%)	0.006
Diabetes	14 (25.0%)	35 (26.3%)	1.00
Chronic kidney disease	17 (30.4%)	44 (33.1%)	0.74
Chronic lung disease	14 (25.0%)	36 (27.1%)	0.86
Congestive heart failure	6 (10.7%)	27 (20.3%)	0.14
Immunocompromised status	32 (57.1%)	83 (61.7%)	0.63
Chronic steroid receipt	16 (28.6%)	31 (23.3%)	0.47
Hematopoietic stem cell transplant recipient	9 (16.1%)	21 (15.8%)	1.00
Heme malignancy	17 (30.4%)	42 (31.6%)	1.00
Autoimmune condition on immunosuppression	7 (12.5%)	12 (9.0%)	0.44
Solid organ transplant recipient	7 (12.5%)	25 (18.8%)	0.40
*Legionella* species (*n* = 70)	*N* = 9	*N* = 63	
*Legionella pneumophila*	0 (0%)	61 (96.8%)	
*Legionella longbeachae*	0 (0%)	2 (3.2%)	
*Legionella maceachernii*	2 (22.2%)	0 (0%)	
*Legionella bozemanae*	3 (33.3%)	0 (0%)	
*Legionella wadsworthii*	1 (11.1%)	0 (0%)	
*Legionella oakridgensis*	1 (11.1%)	0 (0%)	
*Legionella micdadei*	1 (11.1%)	0 (0%)	
*Legionella dumoffii*	1 (11.1%)	0 (0%)	

**TABLE 4:** Clinical course, imaging, and outcomes stratified by *Legionella* PCR melting temperature (Tm, <66°C *versus* ≥66°C)

Variable	Tm <66°C	Tm ≥66°C	*P*-value
Number	56	133	
Computed tomographic imaging	47	121	
Consolidation	39 (83.0%)	113 (93.4%)	0.07
Ground glass infiltrates	44 (93.6%)	118 (97.5%)	0.35
Lymphadenopathy	21 (44.7%)	63 (51.2%)	0.49
Pleural effusion	29 (61.7%)	78 (64.5%)	0.86
Hospital admission	40 (71.4%)	119 (89.5%)	0.004
Hospital length of stay, days (interquartile range)	10 (5–18)	7 (4–15)	0.32
Intensive care unit admission	11 (19.6%)	58 (43.6%)	0.002
Intensive care unit admission length of stay, days (interquartile range)	8 (5–21)	6 (2–12)	0.40
Oxygenation/respiratory support			0.003
Room air	27 (48.2%)	32 (24.1%)	
Nasal cannula	18 (32.1%)	45 (33.8%)	
High flow nasal cannula	2 (3.6%)	4 (3.0%)	
Non-invasive ventilation	1 (1.8%)	2 (1.5%)	
Mechanical ventilation	7 (12.5%)	48 (36.1%)	
Extracorporeal membrane oxygenation	1 (1.8%)	2 (1.5%)	
SOFA II score^a^	1 (1–3.5)	3 (2–5)	0.01
New dialysis	4/54 (7.4%)	22/128 (17.2%)	0.11
Mortality			
In-hospital mortality	9/40 (22.5%)	23/119 (19.3%)	0.65
30-day mortality	9 (16.1%)	23 (17.3%)	1.00
90-day mortality	14 (25.0%)	28 (21.1%)	0.57

^a^ Score assessed for hospitalized patients.

**Fig 4:**
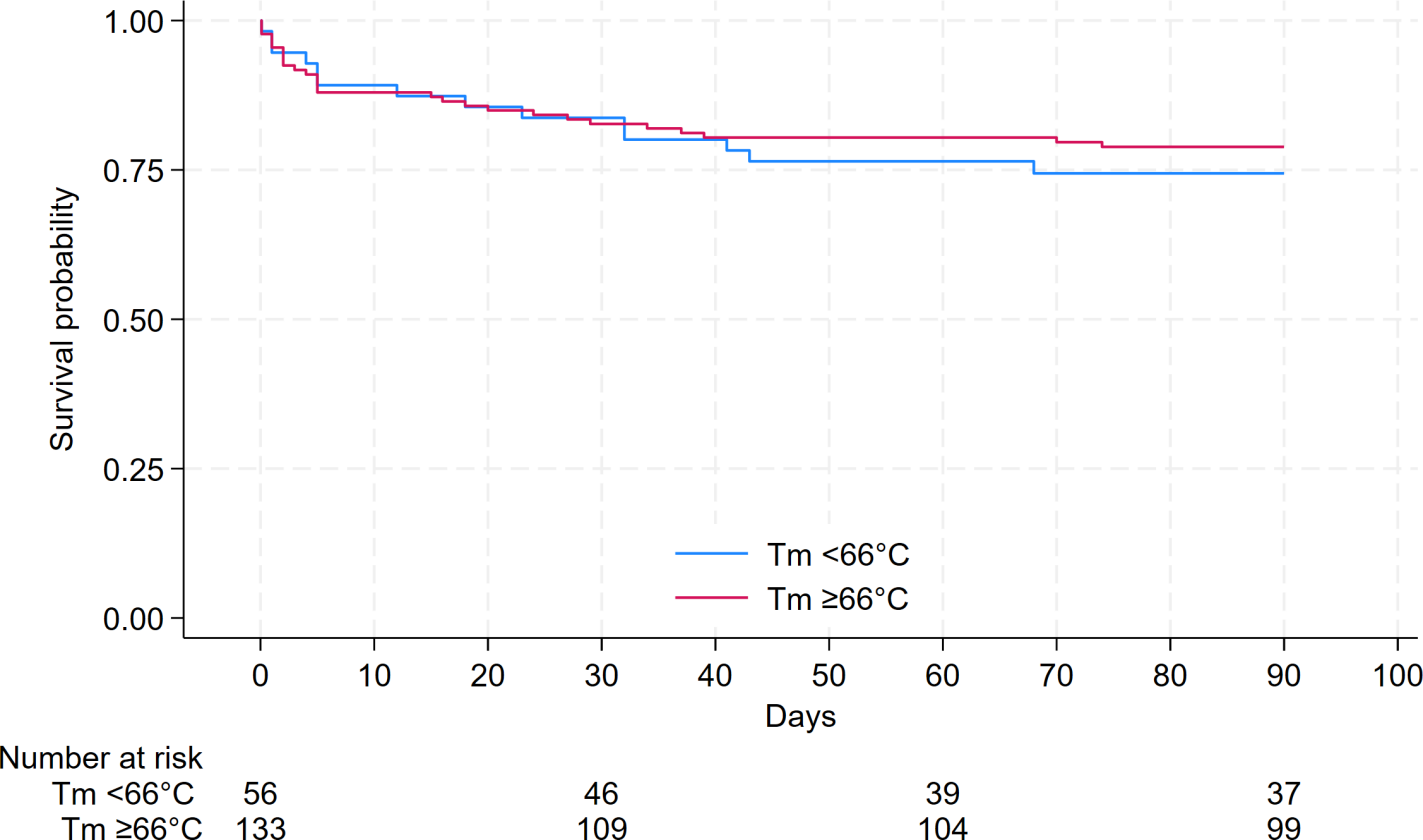
Kaplan–Meier survival curves stratified by *Legionella* PCR melting temperature (Tm, <66°C *versus* ≥66°C).

Ninety-day mortality was 22.2% (42/189). Age was independently associated with mortality (adjusted OR per year 1.04; 95% CI 1.00–1.07; *P* = 0.03), whereas Tm ≥66°C was not (adjusted OR 0.77; 95% CI 0.36–1.67; *P* = 0.51) (Table 5). 36.5% (69/189) were admitted to the ICU. Tm ≥66°C was independently associated with ICU admission (adjusted OR 2.85; 95% CI 1.33–6.11; *P* = 0.007), as was hypertension (adjusted OR 2.10; 95% CI 1.09–4.03; *P* = 0.03), while other variables were not. Kaplan–Meier analysis showed no survival difference between groups (log-rank *P* = 0.56; Fig. 4).

**TABLE 5:** Logistic regression for 90-day mortality and intensive care unit admission

Variable	Univariate odds ratio	95% CI	*P*-value	Multivariable odds ratio	95% CI	*P*-value
90-Day Mortality (*n* = 42/189; 22.2%)						
Age	1.03	1.00–1.06	0.03	1.04	1.00–1.07	0.03
Body mass index	0.99	0.96–1.02	0.66	0.99	0.97–1.02	0.66
Hypertension	1.32	0.66–2.67	0.43	1.34	0.64–2.80	0.43
Immunocompromise	1.42	0.69–2.92	0.34	1.63	0.77–3.45	0.20
PCR melting temperature ≥ 66°C	0.80	0.38–1.67	0.55	0.77	0.36–1.66	0.51
Intensive Care Unit Admission (*n* = 69/189; 36.5%)						
Age	1.00	0.98–1.03	0.71	1.00	0.98–1.03	0.82
Body mass index	1.00	0.99–1.02	0.88	1.00	0.98–1.01	0.77
Hypertension	2.36	1.26–4.42	0.007	2.10	1.09–4.03	0.03
Immunocompromise	0.78	0.43–1.42	0.42	0.71	0.38–1.34	0.29
PCR melting temperature ≥ 66°C	3.16	1.50–6.65	0.002	2.85	1.33–6.11	0.007

## DISCUSSION

In this multicenter cohort, real-time PCR targeting the 5S rRNA gene provided superior detection compared with urinary antigen testing and culture, consistent with prior evaluations of *Legionella* diagnostics (7, 8, 16, 17). Urinary antigen testing, while widely used, primarily detects *L. pneumophila* serogroup 1 and underestimates non-*pneumophila* disease burden, as well as disease burden associated with non-serogroup 1 *L. pneumophila* (7, 17, 18). Here, nearly one-third of PCR-positive cases were consistent with non-*L. pneumophila/*non-*L. longbeachae* species, underscoring limitations of antigen-based testing strategies (7, 17, 19).

PCR substantially outperformed culture, with only 40% of PCR-positive specimens yielding growth. This aligns with published evidence demonstrating the limited sensitivity of culture in routine practice, particularly with low organism burden (7, 16, 20, 21).

A novel observation was the perfect discriminatory performance of Tm analysis (AUC 1.00) for distinguishing *L. pneumophila/L. longbeachae* from other *Legionella* species. Although assay results were clinically reported at the genus level, Tm analysis provides embedded species-level information potentially useful for clinical reporting. Prior work has demonstrated the analytical validity of PCR-based *Legionella* detection (8, 13); the findings presented here extend those to highlight the potential to leverage existing FRET-based data for clinically meaningful species inference. Although inclusivity studies show that *L. gormanii* would also exhibit a Tm ≥66°C, this species is rarely encountered in clinical practice and was not recovered by culture in this study.

Species grouping was independently associated with ICU admission, with Tm ≥66°C detections demonstrating greater odds of requiring ICU care and invasive respiratory support. Severe *L. pneumophila* pneumonia is well described (5, 22, 23); differences in virulence and host–pathogen interaction may contribute to the observed association. However, Tm ≥66°C was not associated with mortality, which was instead primarily driven by age. This aligns with prior data showing that host factors—particularly advanced age and comorbidities—are dominant determinants of survival in *Legionella* pneumonia (5, 12, 22, 23).

Epidemiologic implications are notable. Approximately 30% of infections were attributable to non-*L. pneumophila/*non-*L. longbeachae* species by molecular testing. Reliance on urinary antigen testing alone fails to detect a substantial proportion of *Legionella* infections (7, 17, 19). This may be particularly relevant in immunocompromised populations. It is also especially important in regions where non-*pneumophila* species, such as *L. longbeachae*, are prevalent (7, 19). Environmental and climate influences on *Legionella* epidemiology may also impact detection patterns (24, 25).

Urine *Legionella* antigen testing was performed in 75% of patients in this cohort, reflecting its widespread clinical use. However, its estimated diagnostic yield of 22.5% when used alone underscores its limited sensitivity; a negative urine antigen result should not be used to exclude *Legionella* infection. The findings presented support broader use of PCR as a primary diagnostic modality for suspected *Legionella* infection (7, 18), as well as in those in whom *Legionella* urine antigen testing is negative. Incorporation of validated Tm-based interpretive comments into laboratory reports might enhance epidemiologic surveillance and highlight higher-risk species detections. Notably, non-*L. pneumophila* species identified by melting-temperature analysis would not be detected by assays limited to *L. pneumophila*, including the BIOFIRE FILMARRAY Pneumonia (PN) Panel.

Diagnostic test selection in this cohort included broad use of urine antigen testing. Bronchoscopy—and subsequent PCR and culture-based testing of BAL fluid—was most frequently performed in immunocompromised patients. In our prior analysis, 71.6% of immunocompromised patients underwent bronchoscopy compared with 33.9% of immunocompetent patients (*P* < 0.001), increasing the likelihood of PCR testing in this population (12). As a result, patients undergoing PCR testing were more likely to be immunocompromised and subject to heightened diagnostic scrutiny, which could have introduced selection bias in observed associations.

Limitations include the retrospective design, that all *Legionella* tests were not performed on all patients, and the lack of culture confirmation of all cases. Additionally, PCR detects nucleic acid and does not independently confirm viable infection, raising the possibility of detecting nonviable organisms or contaminants (i.e., lack of specificity). Further, species-level identification based on Tm was not reported clinically and therefore did not directly influence treatment decisions in this cohort.

In summary, real-time PCR targeting the *Legionella* 5S rRNA gene provides superior *Legionella* detection compared to culture and/or urine antigen testing and allows species discrimination through Tm analysis. While *L. pneumophila/L. longbeachae* (Tm ≥66°C) infections were associated with greater acute illness severity, mortality appeared to be primarily driven by host factors. Leveraging embedded molecular data for species inference may enhance public health surveillance of *Legionella* infections and enable higher-risk species discrimination.

## Data Availability

Composite numerical data on which study calculations and results are based are included within the manuscript. Clinical metadata and individual patient results are not publicly available due to institutional policies and patient privacy considerations. Access to these data is subject to Mayo Clinic Institutional Review Board and data governance approval.
